# The Dentist’s Responsibility during the Period of Gestation*Read before the California State Dental Association, June 29, 1921.

**Published:** 1922-03

**Authors:** Arthur H. Nobbs


### THE DENTIST'S RESPONSIBILITY DURING THE PERIOD OF GESTATION *

BY ARTHUR H. NOBBS, B.A., D.D.S.

At first glance one might conclude that the dentist had little responsibility at this particular time. In the few minutes at my disposal I am going to try and establish the fact that it is absolutely possible to lower the infant and maternal death rates by giving proper attention to

* Read before the California State Dental Association, June 29, 1921.

the mother during the period of gestation, and that the members of the dental profession are charged with no small responsibility in the matter. I am also going to point out several ways in which you as dentists can contribute your assistance in this work.

By infant mortality we mean the number of deaths among infants under one year of age for every 1,000 living births. I do not need to emphasize the statement that when we note such a marked variation in the infant mortality rates as is shown in the 1918 report from Ceylon, with a rate of 187.9 per 1,000, and that of New Zealand during the year 1917, with a rate of 48.2 per 1,000, there must be a very positive explanation. I also do not need to explain the statement that from an economic and sociologic standpoint it is very important to control the death rate among infants. We have a fairly good index of the social progress of a community in its mortality tables.

In attempting any work along the lines of antenatal hygiene, there are three factors to consider. The first one is the sociologic factor, which recognizes the question of housing, proper food, sanitation and occupation as affecting the expectant mother. Our ever-increasing number of social workers can take care of this part of the problem.

The second factor is one that is of interest almost entirely to the obstetrician and considers the prevention of birth traumatism, the estimation of the pelvic capacity and the conduct of labor. The last phase of the question is the one that is of particular interest to both dentists and physicians. It is a consideration of the eugenics of the problem. It includes the care of the mother and fetus from the standpoint of prevention of infections and toxemias, the recognition and treatment of disease, and the prophylactic measures that can be instituted in this treatment.

In order to establish the fact that the death rate among infants can be controlled, I am going to quote certain statistics. Most of the figures that I present are taken from the 1919 report of the Department of Commerce, Bureau of the Census. This report is made up of the registration area, which comprises 24 states, representing 58.6% of the population.

During the year there were 1,373,438 live births registered. The mortality rate for infants under one year was 87 per thousand births. There were 119,000 babies who died before they were one year of age, and 47.8% of the total number died before they were one month old. From these statistics we note that one baby out of every twelve dies before it reaches the age of one year. During the same year, in the Australian Commonwealth, the death rate for infants was 69.2 per 1,000. In 1917, the rate in New Zealand was only 48.2 per thousand. It is generally known that the antenatal work in New Zealand is perhaps better organized and conducted, through the activities of the Pluckett Nurses, than in any other country of the world, and this accounts for the very low infant death rate. The United States ranks eleventh among the twenty-six principle nations of the world in infant mortality.

In the state of California the rate is 70 per 1,000. During the year 1919, 3,985 babies in this state died before they were one year of age. In San Francisco, the rate was 63 per 1,000, yet in that same year 515 babies less than one year old were not able to survive the struggle. And even though 63 out of every 1,000 babies born in this city died before they were a year old, we had the lowest infant death rate of any city in the registration area with a population of 250,000 and over during 1919.

The majority of babies who die before they are one month old die because of conditions affecting the health and welfare of the mother, and this fact proves most emphatically that our efforts to control and improve the situation must begin during the period of gestation.

Antenatal hygiene is as important from the standpoint of the mother as it is from that of the child. The death rate of expectant mothers in our country is appalling, and it is estimated that every year 33,000 women die from conditions associated with pregnancy and childbirth. We have a higher maternal death rate than any of the principal countries of the world except Spain and Switzerland.

It is a cold, hard fact that both infant and maternal mortality can be and are being controlled, and the amount of progress made in this direction depends upon the strength of effort made by you and me, by the dental and medical profession and the social workers, and by our legislators. The Shepard-Towner bill now before the Federal Congress will do much to benefit the situation and should have our support.

But these facts and figures only emphasize the point that infant mortality is a controllable condition and that we have a distinct part in the work of prophylaxis. There are other facts concerned with pregnancy that are of particular interest to us as dentists. One of the most important is that of focal infection. Talbot defines a focus of infection as a colony of pathogenic bacteria within the tissues of the body which are in process of active multiplication. Pathogenic bacteria are kept alive by the destruction or digestion of the neighboring tissues of the body. As a result of this breaking down of tissues of the body, we have a bacterial toxin which is not tolerated by the body cells, and which inhibits their function. As a result of this active colony, we may have an absorption of the toxins in the blood stream and thus will be scattered to other parts of the body.

Obstetricians in all parts of the country are considering these foci of infection, which may be ears, teeth, sinuses, tonsils, etc., as a very important factor from the standpoint of prophylaxis against the complications of pregnancy.

In a very hasty survey of the current literature relating to obstetrics, I found ten major pathological conditions of the mother or new-born which were attributed to focal infections, and in each case the obstetrician had made careful observation and study before making his report.

I do not think that the pregnancy of today can be called a true physiological process. Modern habits of dress and diet, as well as the mode of living make the border line very ill defined, and conditions that in the non-pregnant would cause no apprehension, in the pregnant woman may give rise to complications which seriously affect the health of mother and child. This is particularly true in regard to the excretory system. The metabolic processes of pregnant women are much in excess of the normal, and because of this increased activity there is a marked increase in the quantity of waste products which must be excreted by the kidneys and other organs. If the kidney is already taking care of the toxins of focal sepsis in the blood stream, it will be taxed beyond capacity, and a functional failure may result. Therefore the dentist is called upon to remove any foci that may be found in connection with the teeth in order that the strain upon the excretory system may be minimized.

Dental practitioners are often called upon to treat conditions in the mouth that seem to be directly associated with pregnancy. Gingivitis, excessive salivary secretions, loosening of the teeth, hypertrophy of the gum, small areas of ulceration between the teeth, and progressive dental caries are conditions that every one of you with experience has had to deal with. There is much conjecture as to the cause of these conditions. Within very recent years, much investigation has been made by physiologists and biochemists in an effort to find a cause for this pathology, but as far as I know there are only theories and hypotheses offered in explanation. It may be an acidosis caused by defective or impaired metabolism, in which case the cause is a general or systemic one. It may be a local cause, entirely due to changes in the nature of the secretions of the oral glands. In my mind, the judicious and careful practitioner will consider both possibilities and outline his treatment accordingly. Without a doubt, careful and thorough prophylaxis should regularly be given, and the patient instructed as to the proper home care of the mouth.

Carious teeth should be given the proper treatment. It is a false idea that dental operations should not be attempted at this time. The expectant mother needs and deserves your most skillful attention and careful supervision. Any reasonable dental operation can be performed at this time with the usual amount of caution. I believe there is more danger of inducing a premature labor because of a prolonged toothache than if the necessary dental operation for relief was done. Extractions can safely be performed, using any form of anesthesis that is indicated. Nitrous oxid-oxygen anesthesia can be used with little apprehension. It is always best to inquire if the patient has a predisposition to abort. Treatment may have to be modified in that event. One of the biggest tasks that we have to accomplish is to convince our patients that it is not only possible but necessary that they have all abnormal conditions of the mouth and teeth corrected at once.

Your patients will ask what they can do to save their teeth, and to give their baby good teeth. In general, the best advice for them is to live as near normal as possible. Be less scientific and more natural. Eat a sensible variety of the things preferred. Any effort to supply the lime salts by prescribing calcium, etc., will fail, because nature at this time is very particular. She demands certain things, but prefers to select them herself from the ordinary foods. Any neutral mouth wash is good. Lime water has a virtue in that respect, but the calcium in lime water is absolutely non-assimilable by the body, and the bulk of it is excreted, unused. Milk of magnesia may well be used, as the magnesium salts are easily taken up by the blood, and are thus absorbed and redistributed throughout the body. It is claimed by certain biochemists that the solubility of magnesium may help to control the acidosis of the blood.

Calcium is needed by both mother and child for the growth and repair of the skeletal framework. Phosphorus and iron are necessary for the growth of the tissues. These are the important salts to be considered, and a few simple words of advice to the gravid patient will be of much benefit to her.

Without a doubt the demand for mineral salts is greater during the period of gestation than at other times. Provision has to be made not only for the developing tissues of the fetus, but also for changes in the maternal organism. From the laboratory experiments it seems safe to conclude that the developing embryo exhibits a very marked tenacity regarding its supply of calcium. Often the growth of the child seems to be at the expense of the mother. For that reason especial attention should be given to the diet during the period of gestation. All of the lime salts are found combined with the organic substances in food. It is very important that we understand that the simplest way to satisfy the mineral requirements of the body is by advising simple ordinary foods. A saturated solution of lime water contains 1.3 gms. of calcium oxide per pint, and the same quantity of cow's milk contains 1.7 gms. of calcium oxide and, furthermore, the calcium oxide in milk is easily assimilated by the body. One quart of cow's milk per day contains enough calcium oxide to satisfy all the requirements of pregnancy. Green vegetables, fruits, nuts and some dried vegetables also contain calcium salts. Phosphorus and iron can be supplied by advising the use of eggs, cream, cereals, raisins, figs, peas, beans and meats. Orange juice has a high iron content.

In summarizing my remarks, let me again emphasize the point that there is a great need for more antenatal hygiene, and that the dental profession has a distinct place in that work. Your responsibility to your patients who are pregnant is a very definite one and challenges your best study, your most sympathetic interest and all the skill at your command.

BIBLIOGRAPHY

1. Report for year 1919, Births in Registration Area, Department of Commerce, Bureau of the Census.

2. De Lee, A Text Book of Obstetrics.

3. John E. Talbot, "Focal Infection and Its Relation to Obstetrics."

